# Paradoxical relationship between speed and accuracy in olfactory figure-background segregation

**DOI:** 10.1371/journal.pcbi.1009674

**Published:** 2021-12-06

**Authors:** Lior Lebovich, Michael Yunerman, Viviana Scaiewicz, Yonatan Loewenstein, Dan Rokni

**Affiliations:** 1 The Edmond and Lily Safra Center for Brain Sciences, The Hebrew University, Jerusalem, Israel; 2 Department of Medical Neurobiology, School of Medicine and IMRIC, The Hebrew University of Jerusalem, Jerusalem, Israel; 3 The Alexander Silberman Institute of Life Sciences, The Hebrew University, Jerusalem, Israel; 4 Department of Cognitive Sciences and The Federmann Center for the Study of Rationality, The Hebrew University, Jerusalem, Israel; University of Oxford, UNITED KINGDOM

## Abstract

In natural settings, many stimuli impinge on our sensory organs simultaneously. Parsing these sensory stimuli into perceptual objects is a fundamental task faced by all sensory systems. Similar to other sensory modalities, increased odor backgrounds decrease the detectability of target odors by the olfactory system. The mechanisms by which background odors interfere with the detection and identification of target odors are unknown. Here we utilized the framework of the Drift Diffusion Model (DDM) to consider possible interference mechanisms in an odor detection task. We first considered pure effects of background odors on either signal or noise in the decision-making dynamics and showed that these produce different predictions about decision accuracy and speed. To test these predictions, we trained mice to detect target odors that are embedded in random background mixtures in a two-alternative choice task. In this task, the inter-trial interval was independent of behavioral reaction times to avoid motivating rapid responses. We found that increased backgrounds reduce mouse performance but paradoxically also decrease reaction times, suggesting that noise in the decision making process is increased by backgrounds. We further assessed the contributions of background effects on both noise and signal by fitting the DDM to the behavioral data. The models showed that background odors affect both the signal and the noise, but that the paradoxical relationship between trial difficulty and reaction time is caused by the added noise.

## Author summary

Sensory systems are constantly stimulated by signals from many objects in the environment. Segmentation of important signals from the cluttered background is therefore a task that is faced by all sensory systems. For many mammalians, the sense of smell is the primary sense that guides many daily behaviors. As such, the olfactory system must be able to detect and identify odors of interest against varying and dynamic backgrounds. Here we studied how background odors interfere with the detection of target odors. We trained mice on a task in which they are presented with odor mixtures and are required to report whether they include either of two target odors. We analyze the behavioral data using a common model of sensory-guided decision-making—the drift-diffusion-model. In this model, decisions are influenced by two elements: a drift which is the signal produced by the stimulus, and noise. We show that the addition of background odors has a dual effect—a reduction in the drift, as well as an increase in the noise. The increased noise also causes more rapid decisions, thereby producing a paradoxical relationship between trial difficulty and decision speed; mice make faster decisions on more difficult trials.

## Introduction

Natural scenes are cluttered and sensory systems must be able to parse these scenes to detect and identify individual objects such as food, a mate, or a predator. Scene segmentation is critical for all sensory modalities yet the neural mechanisms that underlie this feat are not very well understood. While these mechanisms have been extensively studied in the visual and auditory modalities [1–7], very little is known about scene segmentation of olfactory scenes [8–11].

We have previously developed a psychophysical paradigm for testing detection of target odors against background mixtures in mice [10]. Utilizing this paradigm, we found that the ability to report whether a target odor is present decreases when the number of background odors is increased. Additionally, we found that the overlap between the glomerular activation patterns that represent the target, and those that represent the background odors, was inversely correlated with success rate. This result suggested that background odors may interfere with target-odor detection already at the level of olfactory receptors. Indeed, many studies demonstrated non-linear interactions between odorants activating the same receptor [12–19], and recent studies suggest such interactions are widespread [3,20,21]. However, it is currently unclear how these interactions affect target odor detection and in what way do they interfere with the target signal.

The drift-diffusion model (DDM) has been instrumental in providing insights into the effects of sensory signals on success rate and reaction times in sensory-guided tasks [3,22–27]. According to the DDM, the decision process is determined by a noisy decision variable, which reflects the difference between the accumulated evidence in favor of choosing each of the two alternatives (in this case, the presence *vs* the absence of the target odor). The decision is made once the decision variable reaches one of two thresholds, ±*θ*, where *θ*>0, for the first time. If the decision variable first reaches one of them (*θ*), the decision is to report that the target is present whereas if it first reaches the other (−*θ*), it reports that the target is absent.

The DDM is characterized by several parameters: the first parameter is the starting point *mθ* (−1<*m*<1), which denotes an a-priori, evidence-independent preference towards one of the outcomes. *m* = 0 indicates an unbiased starting point. An a-priori preference towards reporting that the target is present or absent manifests as a positive or negative value of *m*, respectively. The second parameter is the threshold *θ*>0. The larger the value of *θ*, the more evidence is required in order to reach a decision. Third is the average rate of evidence accumulation in the presence and in the absence of the target, which we denote by *A*_+_ and *A*_−_, respectively. We assume that in absence of any odors, $A_{-}^{0}<0$, which biases the decision variable in the direction of the −*θ* threshold and the decision that the target is absent. By contrast, when the target odor is presented to the animal, $A_{+}^{0}>0$ and as a result, the decision variable drifts (on average) towards the *θ* threshold. Finally, the decision variable also accumulates white noise in the decision process, and the magnitude of this noise is denoted by $c_{0}^{2}$ [3,22,27–30].

In this framework, there are at least three different mechanisms by which background odors can interfere with target odor detection. First, they may increase the evidence in favor of the target, thus generating a false target-like signal even when the target is absent, resulting in an increased false-alarm rate. Second, they may reduce the evidence in favor of the target, thereby decreasing the hit rate. Finally, they may act by increasing the noise in the decision-making process. Here we analyze the predictions of the DDM for each of these modes of interference and compare them to experimental observations. We then fit the DDM to the behavioral data to assess the contributions of background effects on signal and noise in the decision-making process.

## Results

**Fig 1:**
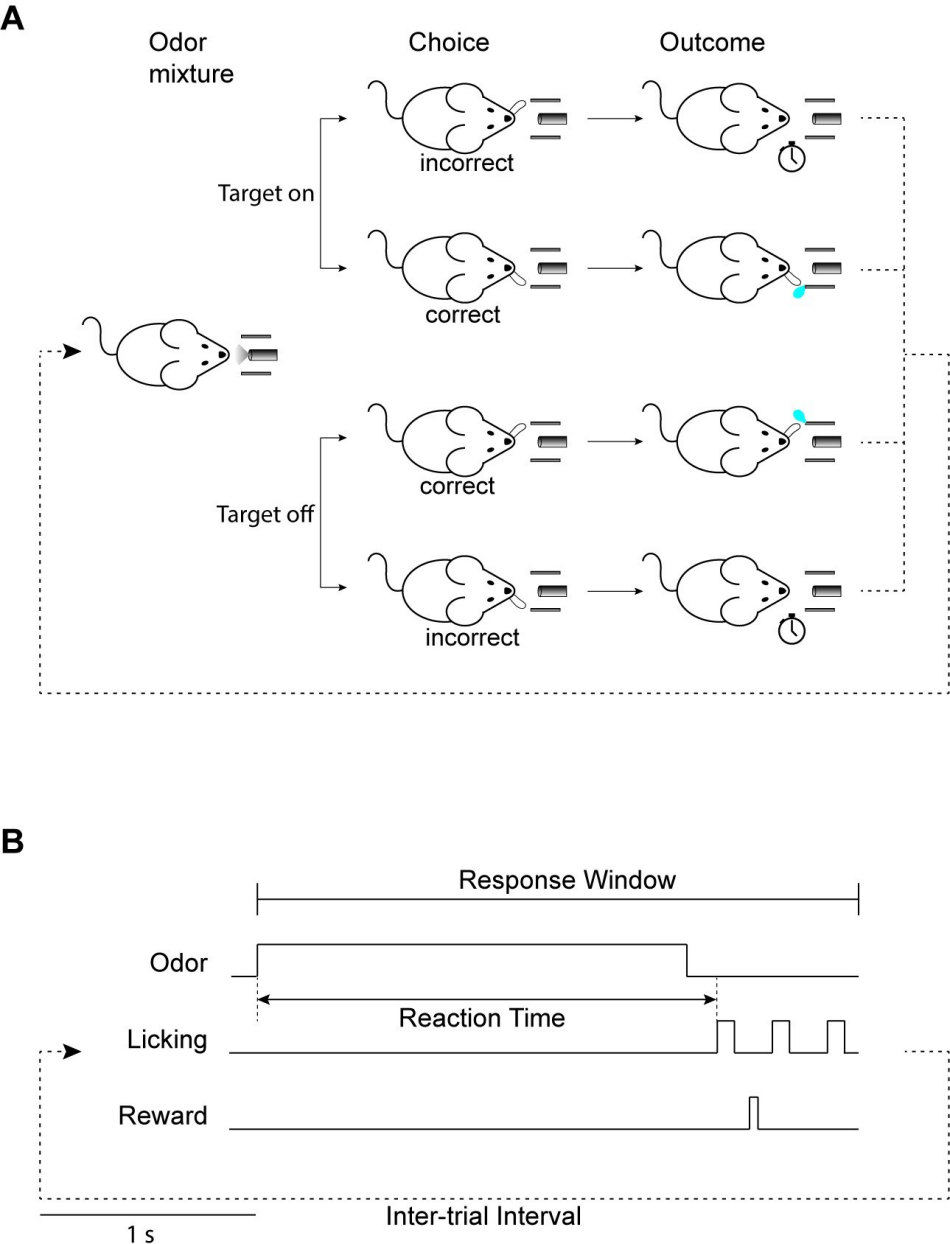
Behavioral paradigm. **A.** Mice were trained on a target detection with background task that was set as a two-alternative choice reaction time task. In each trial, mice were presented with an odorant mixture and were required to report the presence or absence of a target odorant by licking the right or left lick ports, respectively. Correct licks were rewarded with a water drop and incorrect licks were punished with a timeout. **B.** The temporal structure of the task. In each trial an odor mixture was presented for 2 seconds. Mice were required to report their decision by licking within a 2.8 seconds response window that begins with odor presentation. Correct licks were rewarded immediately. The inter-trial interval was 7.2 seconds following correct responses and 12.2 seconds following incorrect responses to encourage accuracy over speed. The time between odor onset and the first lick was taken as the trial’s reaction time.

We trained mice to detect target odorants against background mixtures. The task is similar to the one previously used [10], but modified from a go/no go to a two-alternative choice reaction time design to allow analysis of reaction times in all trials. In each trial, mice were presented with a pseudorandom odorant mixture. Mixtures either included one of two target odorants (“target-on” trials), or neither of the target odorants (“target-off” trials). Mice were rewarded with a water drop if they correctly reported whether the mixture contained a target odorant by licking to the right in target-on trials and licking to the left in target-off trials (Fig 1). To avoid motivating rapid responses, the inter-trial interval was independent of response time, but was 5 seconds longer following incorrect responses. The rate of rewards was therefore dependent on the correct rate, but not the speed of behavioral responses. We trained 6 mice and collected an average of 16790 trials per mouse (range 12000–23000).

We considered three different mechanisms by which background odorants can affect decisions in the framework of the DDM and studied their behavioral predictions:

1. False signal: Individual odors activate a large number of olfactory receptors. It is most likely that many of the target-activated receptors are also activated by the background odors [10]. Therefore, it is possible that the addition of background odorants to the odor mixture will manifest as an *increase* in the drift rates both in the presence of a target odor (*A*_+_) and in its absence (*A*_−_). The larger the number of background odors, the larger the drift rates would be.
2. Signal reduction: Background odors, however, also activate olfactory receptors that are not activated by the target odors. Since inhibitory interactions are common in second and third order olfactory brain regions [31–38], addition of background odorants may inhibit target-associated signals. Such interactions are predicted to manifest as a *decrease* in the drift rates both in the presence of a target odor (*A*_+_) and in its absence (*A*_−_). The magnitude of the decrease would be a monotonic function of the number of background odors.
3. Noise boost: Background odors may affect the dynamics of neuronal responses to the target odor without a consistent effect on mean response. In that case the addition of background odorants could manifest as an increase in the noise associated with the decision process, *c*^2^.

**Fig 2:**
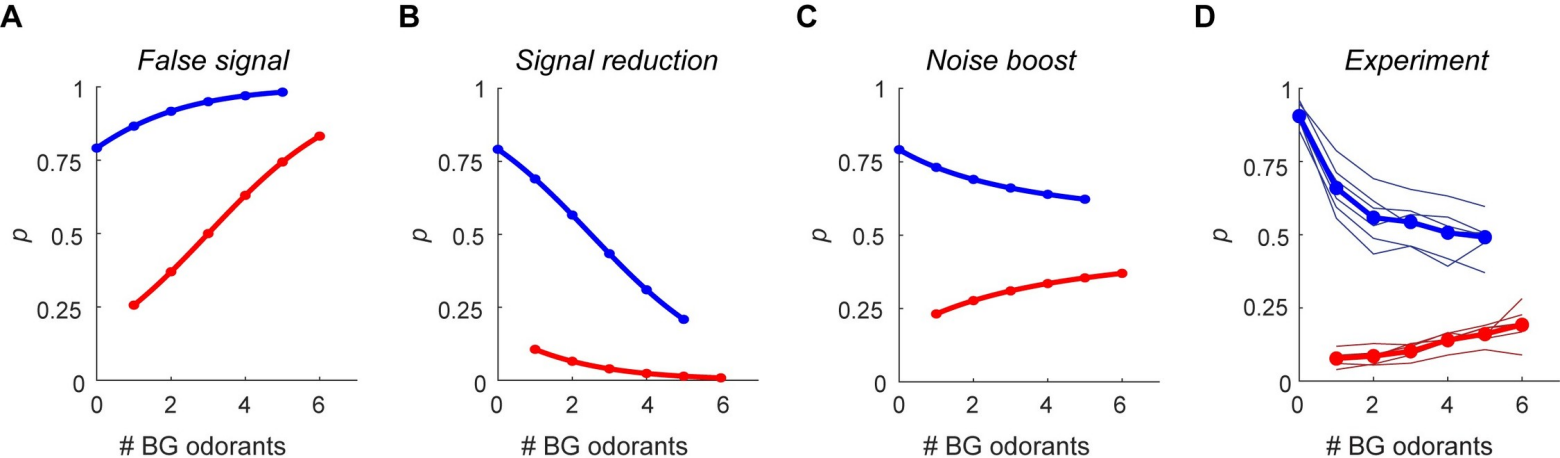
The effect of background odorants on decision probabilities. **A-C.** The probability of reporting that the target odorant is present (*p*) (see Eq 2, Methods) as a function of the number of background odorants, for the three interference hypotheses: false signal (**A**), signal reduction (**B**), and noise boost (**C**). Blue–“target on” trials, red–“target off” trials. Baseline parameter values are *θ* = 1; *m* = 0; $A_{+}^{0}=2;\;A_{-}^{0}=-2.5;\;c_{0}^{2}=3$. The drift rate under the false signal, the signal reduction and the noise boost mechanisms are: $A_{i}=A_{i}^{0}+k∙\#BG,\;A_{i}=A_{i}^{0}-k∙\#BG$ and $A_{i}=A_{i}^{0}$, respectively, where *i*∈{+,−} and #*BG* is the number of background odorants in the mixture and *k* = 1. The diffusion is $c^{2}=c_{0}^{2}+k∙\#BG$ under the noise boost mechanism and $c^{2}=c_{0}^{2}$ in the false signal and signal reduction mechanisms. **D.** The fraction of trials that mice reported that the target is present as a function of the number of background odorants. Thin lines show data from individual mice. Thick lines show the mean across mice. Colors as in A-C.

The behavioral predictions of the three hypotheses are depicted in Fig 2A–2C. These are given by Eq 2 with a linear function on the relevant model parameters (see Fig 2 legend), while the robustness of these results to the model parameters and any monotonous function is given by Eq 3 and 4 (see Methods). The “false signal” hypothesis predicts that an increase in the number of background odors will result in an increase in target detection probability, but will also manifest in an increase in the number of false detections (Fig 2A). In other words, the probability that the subject would report that the target is present *p* would increase with the number of background odorants, both when the target is present (blue) and when it is absent (red). In contrast, the “signal reduction” hypothesis makes the opposite prediction—the probabilities of both true and false detections will decrease with the number of background odors (Fig 2B). Finally, the “noise boost” hypothesis predicts that with the increase in the number of background odorants, choice behavior would contract to a stimulus-independent probability ((*m*+1)/2) resulting in both decrease in the probability of target detection and an increase in the probability of false detection (Fig 2C). Importantly, these predictions are generic to this model and do not depend on a specific choice of parameters (as long as $A_{-}^{0}<0<A_{+}^{0}$) or on the identity of the corresponding monotonic function on the backgrounds. The experimental relationship between the number of background odorants and *p* for both trial types is depicted in Fig 2D (thin lines, single subjects, thick lines, average over subjects). *P* decreased with the number of background odorants in target-on trials, and increased with the number of background odorants in target-off trials. This dependence is only consistent with the prediction of the “noise boost” hypothesis and is inconsistent with the “false signal” and “signal reduction” hypotheses.

**Fig 3:**
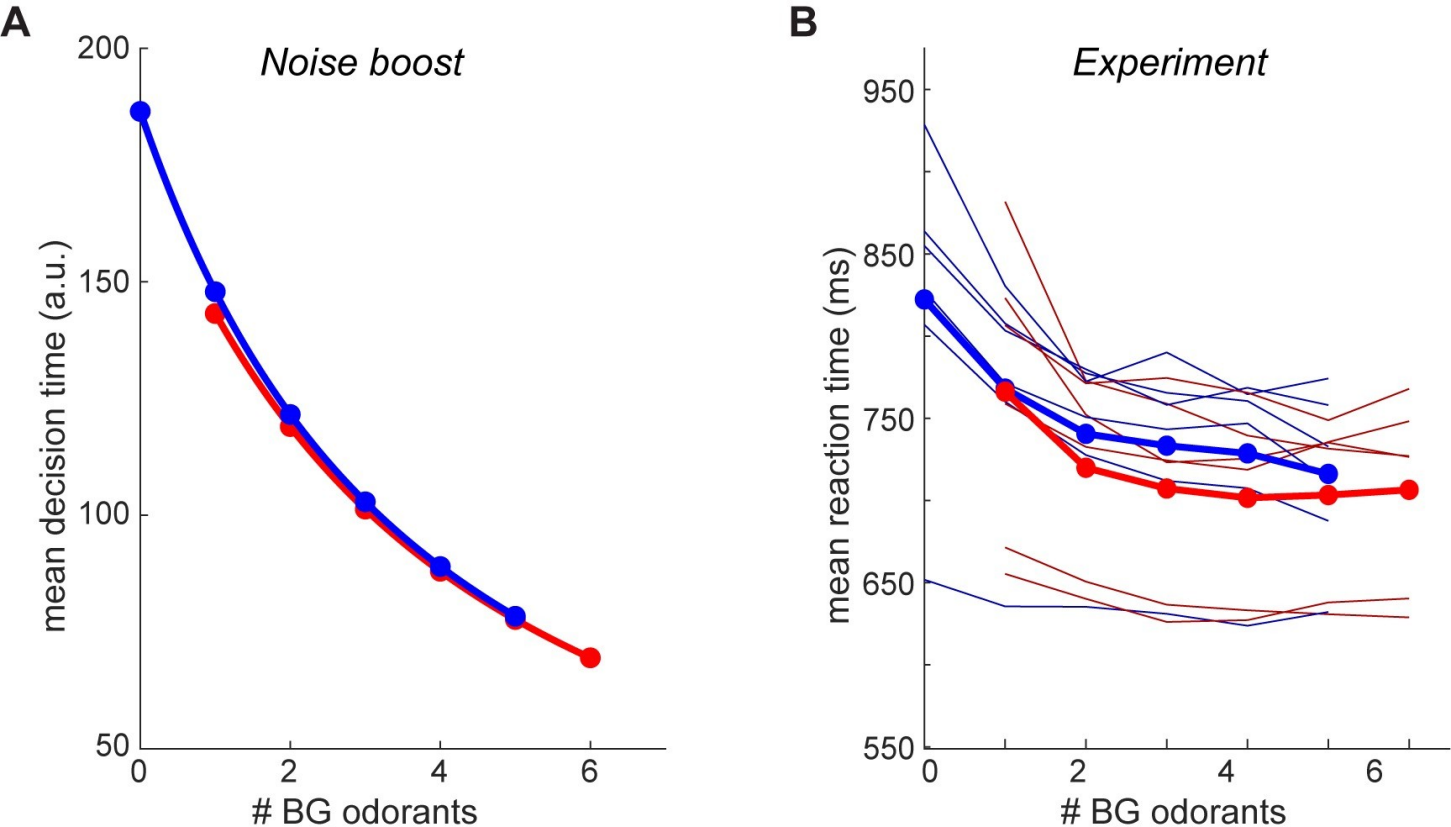
The effect of background odorants on decision times. **A.** Mean decision time (see Eq 5, Methods) under the “noise-boost” mechanism as a function of the number of background odorants. Blue–“target on” trials, red–“target off” trials. Parameter values are as in Fig 2: *θ* = 1; *m* = 0; $A_{+}^{0}=2;\;A_{-}^{0}=-2.5$, where *i*∈{+,−} and $c^{2}=c_{0}^{2}+k∙\#BG$, where #*BG* is the number of background odorants in the mixture and *k* = 1. **B.** The mean mouse reaction time as a function of the number of background odorants. Thin lines show data from individual mice, and thick lines show the mean across mice. Colors as in A.

The “noise boost” hypothesis predicts that the average decision time (Eqs 5 and 6, Methods) would decrease with the number of background distractors both in target-on and in target-off trials (Fig 3A, for the robustness of these results see Eq 6, Methods). To gain insight to this prediction, consider the limit in which the variance of the noise in the decision process is very large. Because the decision variable integrates this noise, the magnitude of the decision variable will very quickly become very large, and will cross one of the decision thresholds. This prediction is counter-intuitive because it suggests that decisions in the more difficult trials–trials associated with more background odorants and hence more errors–- will be made faster. To test this prediction, we measured the speed at which behavioral responses were made (reaction time). Although reaction times include other components beyond decision times, they are useful correlates of decision times. In agreement with the “noise boost” hypothesis, we found that reaction times decreased with the number of background odorants (Fig 3B). This finding is in contrast to a previous study that found a very small effect of background odorants on reaction times, yet that study used a go no go paradigm and was based on a much smaller dataset [10]. Taken together, our results suggest that the effects of background odorants on target detection may be explained by a mechanism in which adding backgrounds manifests as added noise in the decision making dynamics.

Background odorants may reduce target detection by a combined effect on both signal, and noise. To assess the contributions of background effects on signal and noise, we next fitted the DDM to the behavioral data using the chi-square optimization procedure ([39]; also see: Methods). We fit the data of each mouse individually, effectively allowing for both the drift rate and the diffusion to vary freely between the 18 conditions defined by the target identity (target A on, target B on, target off) and the number of background odorants (0–5 and 1–6 for target on and target off trials, respectively; see Methods). One starting point and one *T*_*er*_ parameter, which accounts for the component of the reaction time that is independent of the decision process [39–41], were fit per mouse. Moreover, because the threshold (or, alternatively, the diffusion coefficient, e.g., see: [29]) is a scaling parameter of both the drift and the square root of the diffusion coefficient, the threshold of all mice in all conditions was fixed at the same value (see Methods). Together, 38 parameters were fitted per mouse.

As discussed in the Methods section, the chi-square optimization procedure separately considers 6 RT quantiles in the correct and incorrect trials, resulting in 12 summary statistics per condition per mouse. Thus, the 38-parameter model was fitted using the 12x18 = 216 summary statistics. These summary statistics were based on thousands of trials per animal (average 16,796, range: 12,302–22,954, see also: S1 Fig). On average, there were 933 trials per condition per mouse (16,796/18, range 116–3,439). This variability resulted both from the variability in the number of trials per mouse and from the fact that the number of trials decreased with the number of background odorants (S1 Fig).

**Fig 4:**
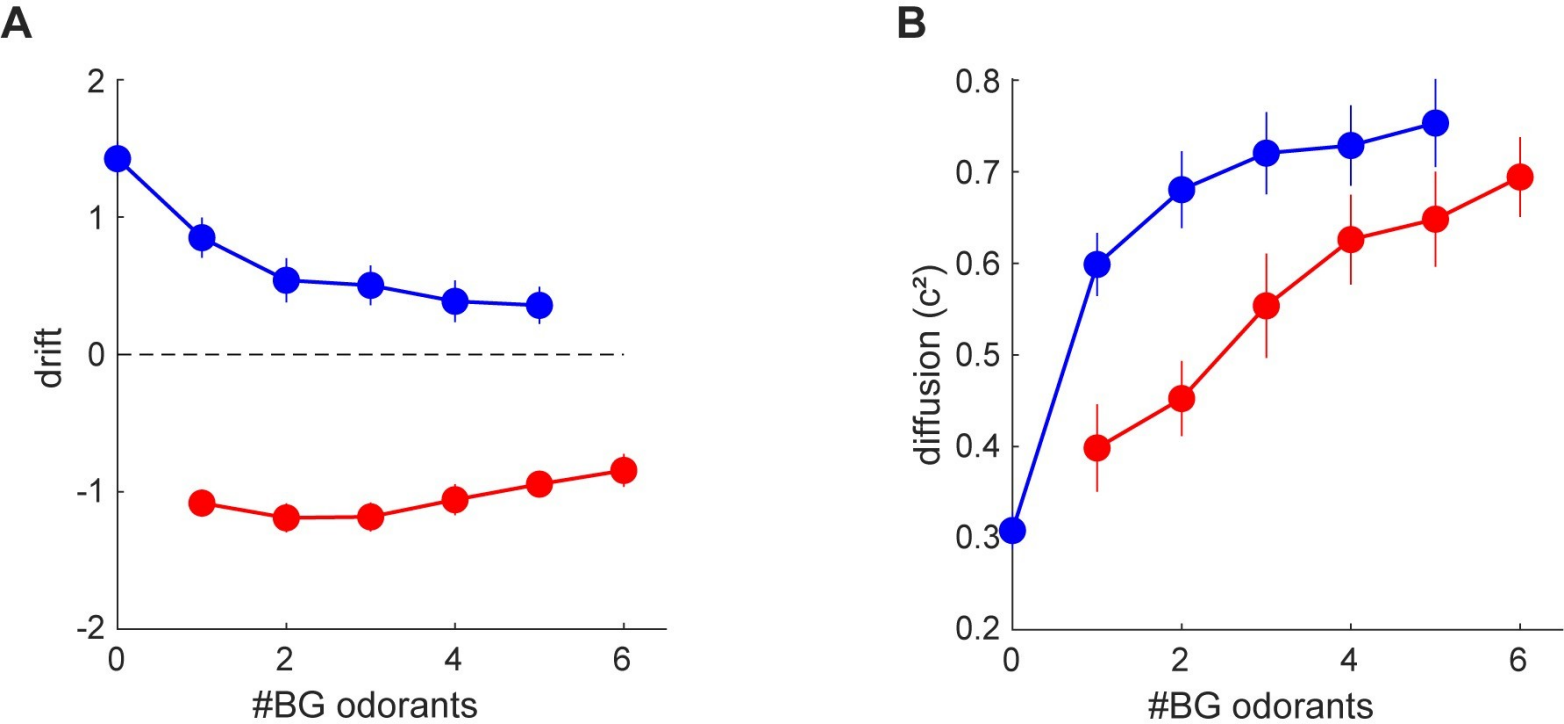
Average drift **(A)** and diffusion **(B)** as a function of the number of background odorants. The plots depict the mean ± SE across mice. Target-on trials are shown in blue and target-off trials in red.

The fitted parameters captured well the relationships between choice probabilities and the number of background odors, as well as the relationships between reaction times and the number of background odors (S2 Fig). Investigating these models revealed that background odors had a consistent effect on both the drift and the diffusion of the decision variable (Fig 4). The absolute value of the drift, consistently decreased with the number of background odors (Fig 4A). Conversely, the magnitude of the diffusion consistently increased with the number of background odors (Fig 4B). These effects were also evident at the level of individual mice (S3 Fig). Both of these effects contribute to the decrease in performance as background odors are added, however only the added noise can explain the decrease in reaction times, as reduced signals increase rather than decrease reaction times. In summary the fitted models indicate that background odors produce a compound effect on the decision making process by both reducing signal and increasing noise. The increased noise is responsible for the paradoxical relationship between trial difficulty and reaction time.

## Discussion

We used the DDM in order to understand how background odors affect the detectability of target odors. We first analyzed the prediction of three simple alternative mechanisms by which background odors may act: inducing target-like signal (‘false signal’), reducing the target signal (‘signal reduction’) or increasing noise (‘noise boost’). The finding that mice make faster and less accurate decisions on trials with more background is only consistent with the increased noise hypothesis. Fitting the DDM to the behavioral data suggests that background odorants act by a combined effect on both signal and noise, but that the paradoxical relationship between trial difficulty and reaction time is caused by the effect on noise.

While our results are general and do not depend on the assumption of specific model parameters and the corresponding (monotonous) function on the backgrounds (see Methods), two limitations of our analysis are worthwhile noting. First, our analysis implicitly assumed that all background odors have the same effect, ignoring their identity, as well as the identity of the target odors. Chemical and representational overlap between the target and background odorants were shown to determine background potency [10]. The magnitude of the background odorant effect may therefore be odorant dependent. A larger behavioral dataset may allow further separation of trials based on specific target-background combinations. Second, our analysis is fully based on a specific theoretical framework–the DDM.

Importantly, our study is, to our knowledge, the first to find an inverse relationship between trial difficulty and reaction time in an odor-guided task. Previous studies used odor discrimination tasks to analyze the relationship between speed and accuracy [42–46]. In such tasks the difficulty is presumed to be related to the signal (odor similarity), and yet contrasting results regarding the effect of discrimination difficulty on reaction times were reported. It is not entirely clear what gave rise to the different results, yet it is possible that the difficulty in some of the tasks that were used was in accurately representing the discrimination boundaries rather than the sensory stimulus itself [46]. Additionally, a recent study indicated that the learning process may also contribute to the relationship between task difficulty, success rate and reaction times [43].

What do our results teach us about cross-odorant interactions? The overlapping nature of sensory representations in the olfactory epithelium provides opportunity for many non-linear odorant interactions [47–51]. Interaction between odorants have been studied mostly at the level of sensory neurons [3,12–19,21] but also, a bit more anecdotally, in the olfactory bulb and cortex [3,52–60]. These studies analyzed the amplitudes of responses to mixtures of odorants and found that these are typically sublinear. The diffusion term in the DDM represents noise that evolves with time within a trial. The finding that background odorants act as noise in the decision making process may therefore suggest that adding background odorants changes not only the amplitude of odor responses but also response dynamics. It will be interesting to analyze the dependence of response dynamics on background in olfactory brain regions.

Background segmentation is a task that is faced by all sensory systems. Is our finding that reaction times decrease with increased background specific to the olfactory system? Although background segmentation has been rather extensively studied in both vision and audition, there are, as far as we know, no studies that immediately compare to ours. When detecting Gabor patterns in noise, the number of eye fixations required for detection increases with various parameters of the stimulus, yet the effects of varying the noise on reaction times have not been examined [3]. Reaction times in tone in noise detection in the auditory system increase when the signal to noise ratio is decreased (either by reducing the signal or enhancing the noise), but the noise in these tasks was continuously present throughout the session and did not obey the trial structure [61,62]. Future experiments that vary the background with a trial structure may promote comparison of the effects of background on reaction times in the different sensory systems.

## Methods

### Ethics statement

All experimental procedures were performed in accordance with the Hebrew University Ethics Committee for Care and Use of Laboratory Animals (approved protocol MD-17-15017-4) and national guidelines.

### Behavior

#### Subjects and surgery

6 c57bl6 adult male mice (10–14 weeks old, Envigo) were trained on the behavioral task. Mice were first anesthetized (Ketamine/Xylazine 100 and 10 mg/kg, respectively) and a metal plate was attached to their skull with dental acrylic for subsequent head restraining. Mice were then housed in pairs and maintained in a reversed light/dark cycle facility. All behavioral training and testing was done during the subjective night time.

#### Apparatus

The behavioral apparatus was located inside a sound attenuating box (Med Associates, VT USA) and consisted of a head restraining device, an odor delivery system, a dual lick detector and a water delivery system. Odor delivery, monitoring of licking and water rewards were controlled using computer interface hardware (National Instruments) and custom software written in LabVIEW. The mouse was continuously monitored using a CCD camera during behavior sessions under red illumination.

#### Odor presentation

Odorant mixtures were presented using a custom-made odor machine as described previously [10]. The odor machine was designed to supply constant flow (1.5 liters/minute) and have the concentrations of the different odorants independent of each other. The odor machine was composed of 8 odor modules. Each module was made of two glass tubes, one containing the odor and solvent and the other containing only the solvent. A 3-way valve (Lee Company, USA) diverted an input flow of filtered air to either the odor tube or the solvent tube, and the output of both tubes was merged to form the module output. This design ensured that each module contributed a constant amount of flow at any time. Input flow to the modules and output flow from the modules were made of ultra-chemical-resistant Tygon/PVC tubing connected in symmetric pair-wise bifurcations to ensure equal flow on all modules. All odorants were diluted to 10% v/v in diethyl phthalate (Sigma Aldrich, CAS 84-66-2) in the tubes and then further diluted in gas phase by the flow of other modules 8 fold. The odorant mixture was carried from the point of final odorant convergence to the odor port through a 1-meter-long tubing with an inner diameter of 1/16 inch to allow mixing while minimizing the latency from valve opening to odor presentation at the mouse’s nostrils. The time delay between valve opening and odor delivery (200 ms), was measured using a photoionization detector (miniPID, Aurora Scietific), and was subtracted from all reaction time values. Odorants were replenished in the vials about once a month to maintain their concentrations.

#### Odor set

All odorants were obtained from Sigma Aldrich. The odorants were (CAS number in parenthesis): Ethyl propionate (105-37-3), Isoamyl tiglate (41519-18-0), Ethyl tiglate (5837-78-5), 2-Ethylhexanal (123-05-7), Propyl acetate (109-60-4), Isobutyl propionate (540-42-1), Ethyl valerate (539-82-2), Phenethyl tiglate (55719-85-2). The target odorant pairs and the mice trained to detect each pair are as follows: Ethyl propionate and Isoamyl tiglate–mice 1&2. Ethyl tiglate and 2-Ethylhexanal–mice 3&4. Propyl acetate and Isobutyl propionate–mice 5&6.

#### Behavioral training and testing

Following surgery mice were allowed one week of recovery and were then water restricted. Mice were then acclimatized to the behavioral apparatus for at least 2 days in which they were allowed 30 minutes of free exploration and free water at the apparatus. This was followed by 2 days in which mice were head-restrained and were rewarded with a water drop for licking the water spouts. To train mice to lick both spouts, drops were delivered randomly at the two spouts. On the fifth day of water restriction mice began training on the task. Mice performed a single daily session of about 1 hour. A mixture was presented for 2 seconds every 10 seconds and mice had to lick to the right if the mixture included one of the two target odorants (target on) and to the left if it did not (target off). Mice had to respond within a 2.8 second period (starting 200 ms after odor onset). Correct licks were rewarded with an 8μL water drop, and incorrect licks were punished by a 5 second time-out. Rejections were not punished and not rewarded. Training began with easy sessions and as mice reached 70% performance over a whole session, session difficulty was adjusted. The difficulty of the task was controlled by varying the distribution of the number of components in the mixture using the following equation:

$$p(x)=\frac{b^{x}}{\sum_{x=1}^{6}b^{x}}$$ (1)

where p(x) is the probability of x components (ranging from 1 to 6). The parameter b was first set to 0.1 and was then raised sequentially through 0.25, 0.5, 0.75 reaching a maximal value of 1. Data was collected only in session with b> = 0.5. Mice performed one session per day with an average of 250 ± 20 trials per session (lasting typically about an hour), and each mouse contributed between 60 and 91 sessions to the data set (72 ± 12 mean ± SD, n = 6 mice). The number of trials each mouse performed under each condition is shown in S1 Fig. Mice often had a bias towards one side (initially often licking to only one side). To eliminate this bias as much as possible, stimulus statistics (target on and target off trials) were made dependent on recent choices. The chance of the next trial being target-on was one minus the proportion of licks to the right in the last 5 trials. Thus if a mouse preferred licking to the left, it would get more trials in which the correct response is to lick to the right. This produced sessions in which the fraction of target on trials was on average 55%. Chance level performance (the performance if mice just guess and ignore the stimulus) is not easily defined here because the bias is not known, however it is always less than 50%.

#### Data analysis

Other than the model fitting (see below), all analysis was performed using custom written code in Matlab. Sessions with complexity below 0.5 were considered training sessions and were not analyzed (except for learning curves). Occasional testing sessions in which performance dropped below 70% (total correct trials/total trials) were also not analyzed (45 such sessions were excluded from a total of 369). All reported parameters were calculated for each mouse separately. To remove between-sessions variation in reaction times that stem from variable positioning of the water spouts, the reaction times in each session were normalized to the mean reaction time in the session. All reaction times were then multiplied by the mean reaction time across all sessions to provide meaningful units. The normalized reaction times and corresponding choices in each trial were used for the analyses below.

### Modeling

According to the DDM, the decision is made once a decision variable *x*, which reflects the difference between the accumulated evidence in favor of choosing each of the two alternatives, reaches one of two thresholds for the first time, ±*θ*, where *θ*>0. Formally, the dynamics within a trial is given by *dx*/*dt* = *A*+*ξ*, where *A* is the drift rate, *t* is time and *ξ* denotes white noise such that E[*ξ*(*t*)] = 0 and E[*ξ*(*t*)*ξ*(*t*′)] = *c*^2^*δ*(*t*−*t*′). The initial state of the decision variable is given by *x*(*t* = 0) = *mθ* (*m*∈(−1,1)). In this model, the probability that the decision variable will reach +*θ* first, *p*, as a function of the model parameters is given by [22,63]:

$$p=\frac{\exp\left(r\right)-\exp\left(-rm\right)}{2∙\sinh\left(r\right)}$$ (2)

where *r* = 2*Aθ*/*c*^2^≠0. We denote by +*θ* and −*θ* the decision thresholds associated with “target present” and “target absent” decisions and by *A*_+_ and *A*− the drift rates associated with trials in which the target odor is present (“target on” trials) and absent (“target off” trials), respectively, and assume that in the absence of background odors, ${A_{+}=A}_{+}^{0}>0,\;{A_{-}=A}_{-}^{0}<0$ and $c^{2}=c_{0}^{2}$.

To study the effect of changing the drift rates or the variance of the noise on *p*, we compute the partial derivative of *p* with respect to these variables:

$$\frac{\partial p}{\partial A}=\frac{\theta ∙g(r)}{c^{2}∙\sinh^{2}\left(r\right)}$$ (3)

$$\frac{\partial p}{\partial c^{2}}=-\frac{A∙\theta ∙g(r)}{{\left(c^{2}\right)}^{2}∙\sinh^{2}\left(r\right)}$$ (4)

where: *g*(*r*) = exp(−*rm*)∙[cosh(*r*)+*m*∙sinh(*r*)]−1.

We note that *g*(*r*), in the domain *D* = {(*r*, *m*)|(−1<*m*<1)∧*r*,*m*∈ℝ}, is bounded from below by inf{*g*(*r*)|(*r*,*m*)ϵ*D*} = 0. This is because the first derivative of *g*(*r*) in *D*, ∂*g*/∂*r* = *e*^−*rm*^∙(1−*m*^2^)∙sinh(*r*) = 0 only for *r* = 0, the value of the second derivative of *g*(*r*) in *D* at $r=0,\;{\partial^{2}g/\partial r^{2}|}_{r=0}={e^{-rm}∙(1-m^{2})∙[-m∙\sinh\left(r\right)+cosh(r)]|}_{r=0}=(1-m^{2})>0$, and hence, *g*_*D*_(*r*) has an absolute minimum at *r* = 0. Thus, *g*_*D*_(*r*)≥*g*(0) = 0. Because ∂*g*/∂*r* is strictly positive for *D*∩{*r*≠0}, *g*_*D*∩{*r*≠0}_(*r*)>*g*(0) and thus *g*(*r*) is strictly positive for *r*≠0 and −1<*m*<1. Therefore, $\frac{\partial p}{\partial A}>0$ and $\frac{\partial p}{\partial c^{2}}=\left\{ \begin{array}{cc} <0 & A>0\\ >0 & A<0 \end{array}\right.$.

According to the “false signal” hypothesis, both *A*_+_ and *A*_−_ are monotonously increasing functions of the number of background odor. Thus, the addition of background odors increases *p*, and hence increases the probability of a correct response when the target odor is present and decreases it when the target is absent (note that the probability of a correct response is *p* in “target on” trials and 1−*p* in “target off” trials). Similarly, the “signal reduction” hypothesis posits that *A*_+_ and *A*_−_ decrease monotonously with the addition of background odors, with the opposite effects on *p*. Finally, in the “noise boost” hypothesis, background odors increase *c*^2^ monotonously without changing *A*_+_ and *A*_−_, which results in a decrease in the probability of a correct response both when the target odor is present and when it is absent.

Next, we consider the effect of odor backgrounds on the speed at which decisions are being made in the “noise boost” mechanism. In the DDM, the mean decision-time, E[*DT*], as a function of the model parameters is given by [22,63]:

$$E[DT]=\frac{\theta ∙[\cosh\left(r\right)-m∙\sinh(r)-\exp\left(-r∙m\right)]}{A∙\sinh\left(r\right)}$$ (5)

The derivative of E[*DT*] with respect to *c*^2^ is given by

$$\frac{\partial E[DT]}{\partial c^{2}}=-\frac{2\theta^{2}∙g(r)}{{\left(c^{2}\right)}^{2}∙\sinh^{2}\left(r\right)}<0$$ (6)

Thus, if *c*^2^ is a monotonously increasing function of the number of background odors, adding background odors is predicted to decrease E[*DT*].

### Quantile estimation of the DDM parameters

The behavioral dataset in this experiment consists of hundreds of trials per mouse per condition (see S1 Fig). Under these circumstances [64,65], we fit the DDM to the response data of each mouse using the Chi-square quantile optimization procedure [39] available through HDDM Python toolbox [66]. We did not use a Bayesian hierarchical procedure, both because of the data abundance but also, because the identity of target odors changed between every 2 pairs of mice, making the fitting of group parameters less informative. Under the Chi-square fitting procedure, the empirical correct and error response data in each condition are separately divided into 6 RT bins, according to 5 quantiles (0.1, 0.3, 0.5, 0.7 and 0.9), and the expected cumulative probability up to each quantile is computed using the theoretical defective cumulative probability. Subtracting the expected cumulative probabilities of each pair of successive quantiles and multiplying the result by the total number of either correct or error trials in that condition results in the expected frequencies corresponding to each of the 12 bins. The objective of this fitting procedure is to minimize the sum of (*O*−*E*)^2^/*E* between observed and expected frequencies, summed over the 12 bins and over conditions. In addition, the fit procedure assumes some portion of uniformly distributed outlier data, which do not follow the DDM dynamics (e.g., due to attentional lapses). As commonly applied by current DDM fitting procedures, we allowed for 5% of responses to be discarded (for more details, see: [39,66].

To assess the contributions of background effects on both signal and noise, we fit the response data of each mouse to the DDM using the above Chi-square procedure. We note that in fitting data to the DDM, the diffusion parameter is commonly viewed as a scaling parameter of the drift and threshold parameters and it is therefore typically fixed at some constant value (frequently 0.1 or 1, [29,67,68]. Equivalently, however, each of these 3 model parameters may be viewed as a scaling parameter of the other two and thus fixed at some constant value in order to measure how the remaining parameters change between conditions. Because we were interested in the effect of backgrounds on both the signal and noise and as we used a custom code, in which the diffusion variance is set to 1 [66], the fitted parameters were scaled to quantify the backgrounds effect on the drift (*A*) and diffusion variance (*c*^2^). The remaining parameters (*m*′ and *T*_*err*_, see below) are not affected by this scaling and thus do not require a similar adjustment. We also note that unlike the DDM presented above, the fitting procedure we used assumes that the upper and lower thresholds and the starting point are located at *θ*′, 0 and 0<*m*′*θ*′<*θ*′, respectively (instead of ±*θ* and −*θ*<*mθ*<*θ*, as in our DDM modeling framework), though it can be shown that setting *θ* = *θ*′/2 and *mθ*+*θ* = *m*′*θ*′ depicts the same process. Thus, the fitted parameters were scaled according to: $A=\frac{A_{fit}}{\theta_{fit}},\;c^{2}=\frac{1}{{\left(\theta_{fit}\right)}^{2}},$ *θ*′ = 1 (*θ* = 1/2) and *m* = 2*m*′−1.

For each mouse and each of the 18 conditions– 3 possible targets modes X 6 possible numbers of background odorant (target A on: 0–5 backgrounds; target B on: 0–5 backgrounds; no target: 1–6 backgrounds)–we allowed the threshold (*θ*′) and drift parameters (*A*′) to vary between conditions, effectively allowing the drift rate (*A*) and diffusion variance (*c*^2^) parameters to vary between conditions. To fit the model to the data, *T*_*er*_ is added to account for the component of the reaction time that is independent of the decision process [39–41]. In our fitting, one non-decision time, *T*_*err*_, and one starting point, *m*′, were fit per mouse and were not allowed to change between conditions. This resulted in an overall of 38 parameters that were fitted per mouse. Figs 4 and S3 depict the adjusted drift rate (*A*) and diffusion variance (*c*^2^) parameters. To evaluate the quality of the DDM fit, we simulated 1000 responses based on the fitted parameters obtained for each mouse and each condition and compared these with the empirical data. The DDM provided reasonable fits to the by-mouse by-condition *p* and median reaction times (see S2 Fig). We note that comparable results were obtained using a Maximum-likelihood fitting procedure (not shown, but see: data and code availability).

## Supporting information

S1 Fig The number of target-on (blue) and target-off (red) trials performed by each mouse for each number of background odorants.
(TIF)

S2 Fig Comparison of experimental and DDM predicted decisions (**A**), and decision times (**B**). Behavioral data (colored) and Model predictions (black) are shown for each mouse (column). Data and model predictions are shown separately for target A trials (blue), target B trials (purple), and no target trials (red). Reaction times are shown as median ± median absolute deviation.
(TIF)

S3 Fig Drift (**A**) and Diffusion (**B**) extracted from the DDMs fit to individual mice. The mean values for each mouse and number of background odors are shown. trials are separated by target odor content: target A (blue), target B (purple), and no target (red).
(TIF)
