# Supplementary figures and images for: Paradoxical relationship between speed and accuracy in olfactory figure-background segregation
Source: PLoS Comput Biol. 2021 Dec 6;17(12):e1009674. doi: 10.1371/journal.pcbi.1009674 (PMC8675919; doi:10.1371/journal.pcbi.1009674)

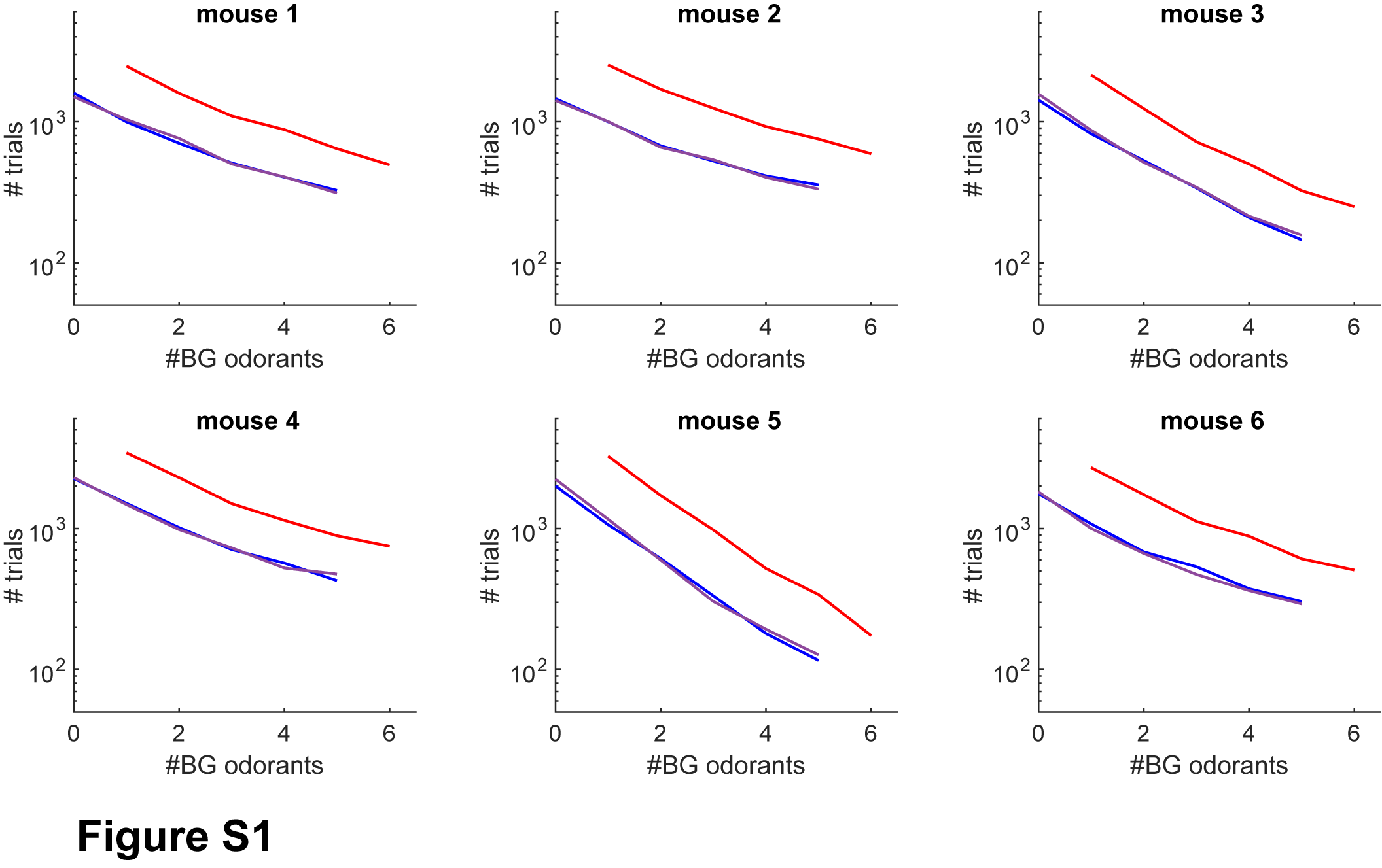

Supplement: S1 Fig — The number of target-on (blue) and target-off (red) trials performed by each mouse for each number of background odorants. (TIF) [file pcbi.1009674.s001.tif]

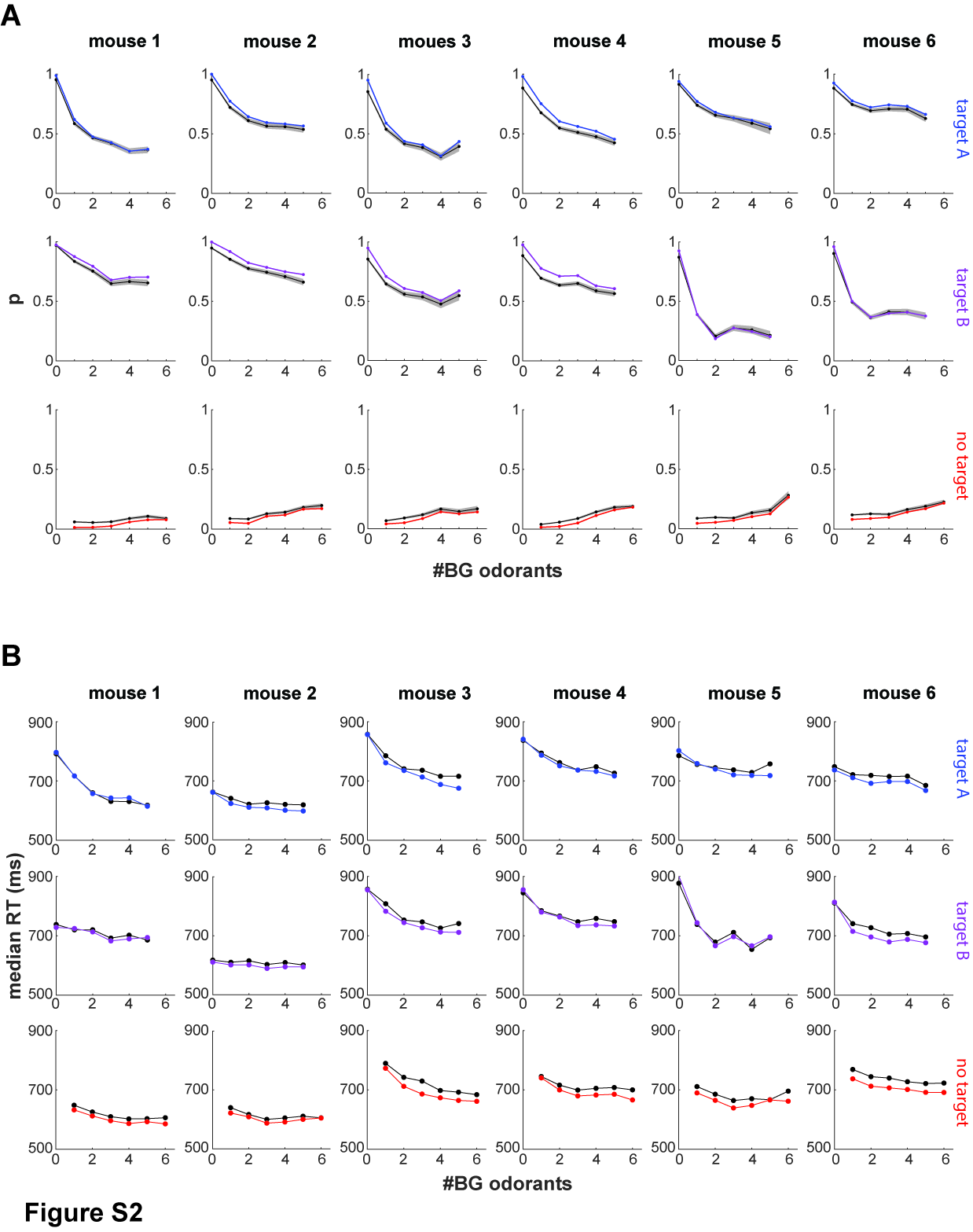

Supplement: S2 Fig — Comparison of experimental and DDM predicted decisions (A), and decision times (B). Behavioral data (colored) and Model predictions (black) are shown for each mouse (column). Data and model predictions are shown separately for target A trials (blue), target B trials (purple), and no target trials (red). Reaction times are shown as median ± median absolute deviation. (TIF) [file pcbi.1009674.s002.tif]

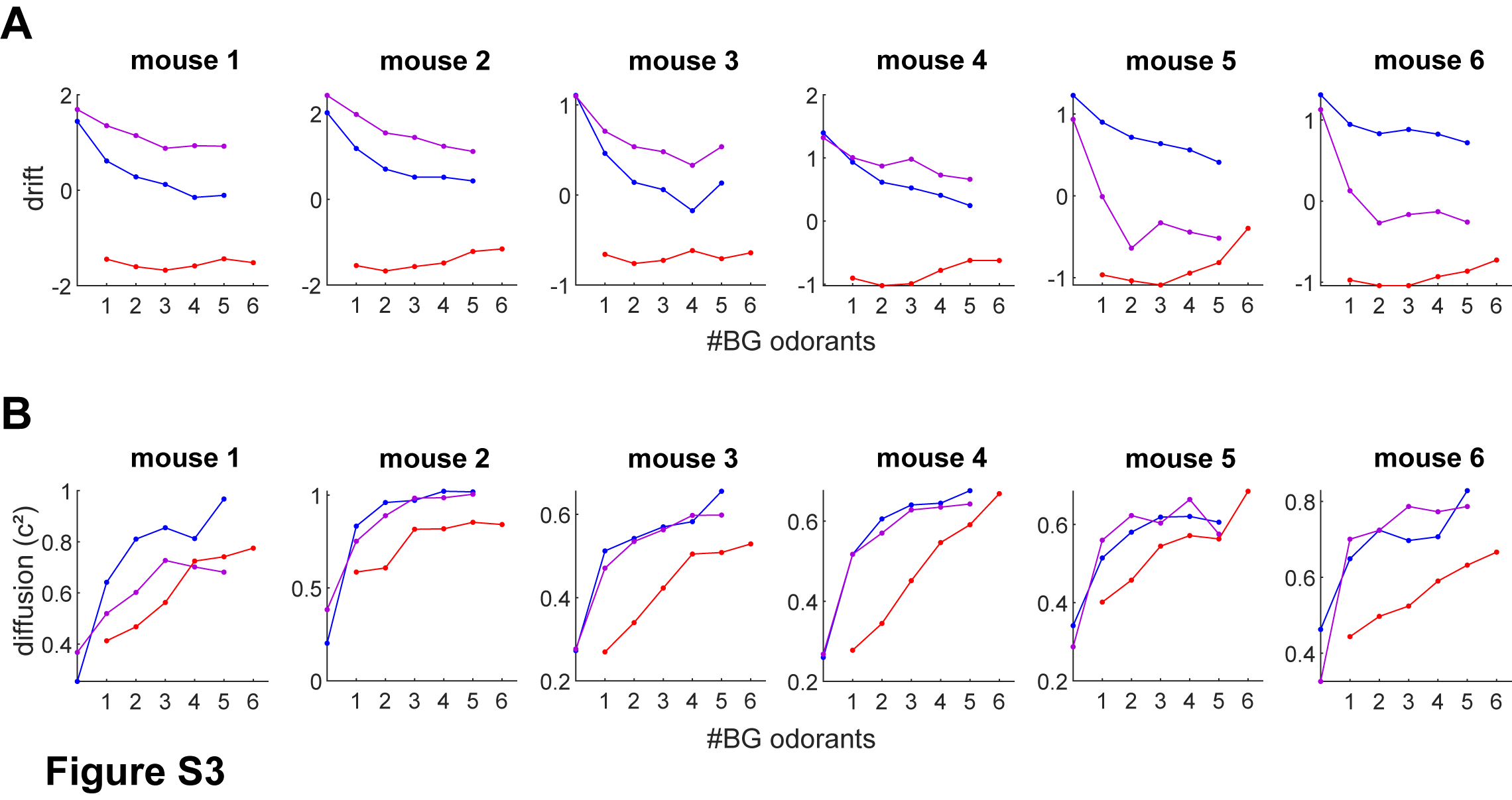

Supplement: S3 Fig — Drift (A) and Diffusion (B) extracted from the DDMs fit to individual mice. The mean values for each mouse and number of background odors are shown. trials are separated by target odor content: target A (blue), target B (purple), and no target (red). (TIF) [file pcbi.1009674.s003.tif]
